# Modern Analytical Chemistry Meets Heritage Books: Analysis of Volatile Organic Compounds (VOCs) from Two Books Preserved at the Biblioteca Capitolare of Busto Arsizio

**DOI:** 10.3390/molecules30112447

**Published:** 2025-06-03

**Authors:** Chiara Chiodini, Pierangela Rovellini, Matteo Chiodini, Luca Giacomelli, Daniela Baglio, the 5B IISS Torno Working Group

**Affiliations:** 1 IISS Torno, 20022 Milan, Italy; 2 Innovhub SSI, 20133 Milan, Italy; 3 Polistudium SRL, 20121 Milan, Italy

**Keywords:** heritage preservation, volatile organic compounds, headspace SPME-GC/MS, paper degradation markers, non-invasive analysis

## Abstract

The development of sensitive, non-invasive methods is essential for the preservation and study of heritage books, allowing insights into their historical production processes and conservation needs. Volatile organic compound (VOC) analysis provides a valuable, non-destructive approach to assess paper composition and degradation in historical volumes. In this study, we analyzed VOC emissions from two books preserved at the Biblioteca Capitolare of Busto Arsizio, Italy: a 16th-century Latin grammar book and a 19th-century mathematics handbook for measurement conversions. Using headspace solid-phase microextraction (HS-SPME) and gas chromatography–mass spectrometry (GC-MS), VOCs were sampled after 24 h of storage at room temperature. The results revealed distinct degradation markers: Straight-chain aldehydes, indicative of lipid oxidation, were more prevalent in the 16th-century book, reflecting the higher quality and durability of its rag-based paper. In contrast, elevated furfural levels in the 19th-century book suggest accelerated cellulose hydrolysis typical of wood pulp paper. Additionally, the presence of menthol and anethole in both volumes points to the use of bacteriostatic agents for preservation. These findings not only highlight differences in material composition but also underscore the importance of tailored conservation approaches for historical documents from different eras.

## 1. Introduction

The preservation of historical paper artifacts, including books and manuscripts, is vital to safeguarding cultural heritage [1,2,3,4,5]. These items embody a tangible connection to past knowledge, artistic practices, and social values, making their conservation essential for maintaining a link to history. However, due to varying materials and production techniques, books from different historical periods face diverse challenges of degradation [1,6]. Monitoring and understanding these degradation processes is therefore critical to developing conservation strategies that extend the longevity and accessibility of these valuable artifacts.

Paper degradation typically occurs through two primary mechanisms: acid-catalyzed hydrolysis and oxidative degradation [7,8,9]. Books made from wood pulp, especially those produced after the 19th century, are susceptible to rapid degradation. The acidic compounds in wood pulp catalyze the breakdown of cellulose fibers, producing byproducts like furfural, a marker of cellulose and hemicellulose hydrolysis. This degradation is associated with a significant decline in paper quality. In contrast, historical papers produced from plant-based rags, as in books from the 16th century, contain fewer acidic components and are thus more resistant to hydrolysis. Instead, these older papers tend to degrade via the oxidation of residual lipids, releasing aldehydes like octanal and nonanal as byproducts, which serve as markers of oxidative degradation.

**Figure 1:**
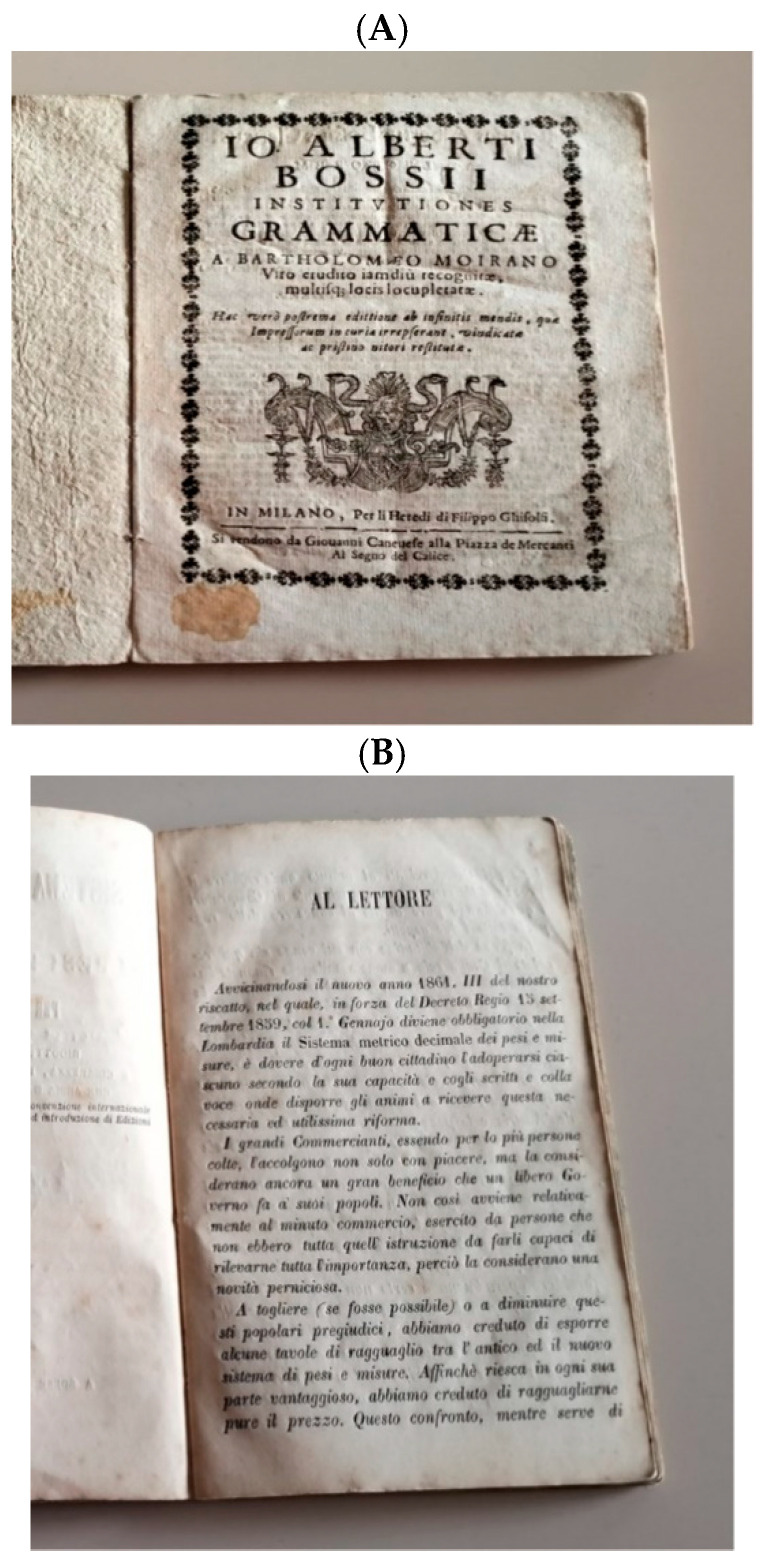
The two heritage books examined in this study. Panel (**A**): XVI century book. Written by Gian Alberto Bossi, this Latin grammar became a bestseller and was widely used by teachers instructing students from noble families. Preserved in Busto Arsizio, it remains relevant today, with examples still used in modern classrooms. Panel (**B**): XIX century book. This 1860 book on mathematical conversions, acquired at an antique market, was widely used in early post-unification Italy to standardize local measurements according to the new national system.

In this study, we analyzed volatile organic compounds (VOCs) emitted from two books preserved at the Biblioteca Capitolare of Busto Arsizio in Italy (Figure 1) [10]. The first volume, a Latin grammar book by Gian Alberto Bossi, was published in the late 16th century and widely used to teach noble families, with its content still relevant in some classrooms today. The second volume, a mathematics handbook published in 1860, was designed to standardize measurement conversions in post-unification Italy. The books represent distinct production materials and processes, offering insights into different degradation pathways and preservation needs.

Headspace solid-phase microextraction (HS-SPME) coupled with gas chromatography–mass spectrometry (GC-MS) enables non-invasive VOC analysis, preserving the structural integrity of valuable documents [9]. This approach allows for the identification of compounds linked to specific degradation mechanisms, such as aldehydes from lipid oxidation and furfural from cellulose hydrolysis. The technique provides conservators and researchers with a means to monitor the chemical health of historical documents, minimizing risk to the artifacts. 

This study aims to identify and compare degradation markers in two heritage books from different centuries to assess their respective preservation states. Using non-invasive HS-SPME/GC-MS analysis, we analyzed the VOCs emitted from a 16th-century Latin grammar book and a 19th-century mathematics handbook preserved at the Biblioteca Capitolare of Busto Arsizio, Italy [9,11]. By examining specific VOC profiles, such as aldehydes and furfural, this research seeks to provide a clearer understanding of the degradation pathways affecting each type of paper, offering insights relevant to conservation science. This collaborative work between IIS G. Torno, Biblioteca Capitolare of Busto Arsizio, and InnovHub SSI highlights the importance of partnerships in heritage science, facilitating the preservation of historical artifacts for future generations.

## 2. Results

The analysis of VOCs emitted from the two historical books revealed distinct profiles that reflect differences in their material composition and degradation pathways. Each book’s VOC profile highlights its preservation state, providing insights into how historical manufacturing techniques influence long-term chemical stability. The main compounds that allow differentiation of the degradation processes are listed in Table 1.

### 2.1. VOC Profile of the 16th-Century Book

**Figure 2:**
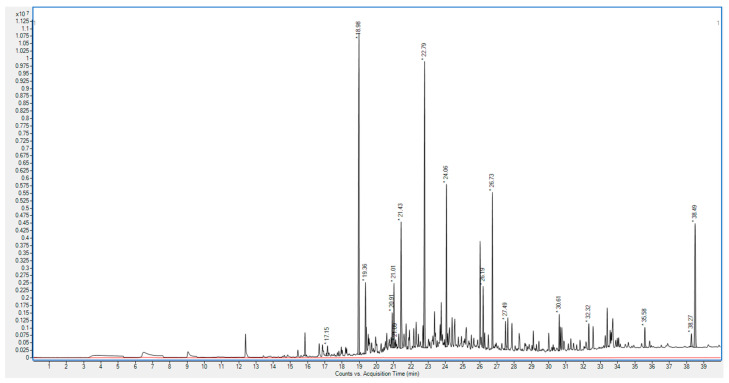
GC spectrum of the VOC profile for the 16th-century Latin grammar book (black line) and blank exposure analysis (red line). Y-axis label: Total ion current.

The 16th-century Latin grammar book exhibited a VOC profile rich in alkanes and aldehydes, which are indicative of lipid oxidation—a slower degradation pathway associated with high-quality rag-based paper (Figure 2) [8,9]. The profile was dominated by tetradecane (12.48% of total VOCs) and hexadecane (10.45%), long-chain alkanes likely originating from residual organic components in the plant-based rag fibers. These alkanes suggest the presence of stable, organic compounds within the paper, contributing to its durability. Additionally, moderate levels of aldehydes, such as nonanal (2.36%) and decanal (4.76%), support the presence of oxidative degradation as the primary pathway, while octanal was detected at a lower concentration of 0.42%.

Furfural, a typical marker of acid-catalyzed hydrolysis, was found in minimal amounts (0.11%), underscoring the rag paper’s chemical resistance to acid degradation [12]. Certain compounds, such as butoxyethoxy ethanol (4.35%), menthol (4.67%), and anethole (1.05%), may suggest historical conservation efforts, although no documentation is available for these specific books. Both menthol and anethole possess bacteriostatic properties, which could hypothetically support such a role, but their presence may also stem from ambient exposure or unrelated sources [13,14]. This VOC profile underscores the durability of the 16th-century book, with a slow, oxidative degradation pathway that aligns with the chemically stable nature of rag-based paper.

### 2.2. VOC Profile of the 19th-Century Book

**Figure 3:**
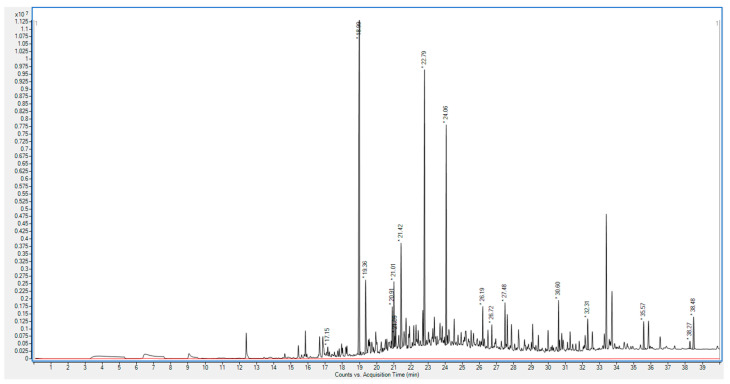
GC spectrum of the VOC profile for the 19th-century mathematics handbook (black line) and blank exposure analysis (red line). Y-axis label: Total ion current.

The 19th-century mathematics handbook displayed a VOC profile that reflects the composition and degradation characteristics of wood pulp-based paper (Figure 3) [8,9]. The profile was rich in long-chain alkanes, with tetradecane comprising 17.81% of total VOCs and hexadecane comprising 10.06%. These alkanes are common in wood pulp paper and likely represent residual organic compounds within its structure. In addition to alkanes, furfural was detected at a concentration of 0.31%, indicating acid-catalyzed hydrolysis, which is prevalent in wood pulp due to its acidic nature.

The profile also included notable levels of menthol (6.30%) and synthetic compounds such as diphenyl ether (1.42%) and diethyl phthalate (1.05%). The higher menthol content suggests possible preservation treatments to counteract microbial damage, while synthetic additives like diethyl phthalate may have been introduced to extend the lifespan of the paper. The presence of furfural and synthetic compounds suggests a more chemically vulnerable paper type, susceptible to rapid degradation under acidic conditions, which may have prompted additional preservation efforts [12].

### 2.3. Comparative Analysis of VOC Profiles and Implications for Conservation

A comparative analysis of VOCs based on peak area integration highlights the distinct degradation mechanisms of each book (Table 1). The 16th-century book exhibited high levels of long-chain alkanes (e.g., tetradecane at 12.48% and hexadecane at 10.45%) and moderate levels of aldehydes (e.g., decanal at 4.76% and nonanal at 2.36%), indicating that oxidative degradation is the primary pathway, consistent with the stability of rag-based paper. The minimal concentration of furfural (0.11%) further supports the chemical resilience of this material against acid hydrolysis, demonstrating the durability of rag-based paper in historical artifacts.

In contrast, the 19th-century book showed a higher concentration of furfural (0.31%), reflecting its vulnerability to acid-catalyzed hydrolysis due to the inherent acidity of wood pulp paper [12]. This increased susceptibility to hydrolytic degradation in the 19th-century book underscores the rapid deterioration that acidic materials face, especially in environments where preservation conditions may fluctuate. The relatively lower levels of aldehydes in this book compared to the 16th-century book suggest that oxidation is a secondary degradation pathway, less significant in the chemically unstable environment created by acidic wood pulp.

These findings are relevant for heritage science as they reveal how historical manufacturing methods and material choices directly impact the longevity of cultural artifacts. The chemically stable nature of rag-based paper used in the 16th-century book suggests that such materials are inherently better suited for long-term preservation, with oxidative degradation occurring at a relatively slow pace. This durability makes rag-based artifacts more resilient to environmental fluctuations and less dependent on rigorous conservation efforts.

Conversely, the findings for the 19th-century book emphasize the inherent vulnerability of wood pulp paper to acidic degradation. The high furfural content highlights a pressing need for targeted conservation strategies, especially for collections dominated by 19th-century and early 20th-century materials [12]. Preservation efforts for such books may require more stringent environmental controls to limit acid hydrolysis, such as maintaining stable humidity levels, reducing temperature fluctuations, and possibly applying deacidification treatments to neutralize the acidic compounds within the paper fibers.

Additionally, the presence of menthol and synthetic compounds in both books points to historical or contemporary preservation interventions, reflecting an ongoing awareness of the need to mitigate microbial damage and extend paper longevity [13,14,15]. These findings underline the role of active preservation treatments and monitoring, especially for vulnerable wood pulp-based collections, and suggest that conservation strategies can benefit from regular VOC profiling to assess the effectiveness of these treatments over time.

## 3. Discussion

This comparative VOC profile demonstrates how historical paper composition and manufacturing methods significantly influence the preservation state of cultural artifacts. The findings advocate for conservation approaches tailored to the specific degradation mechanisms of each paper type, ensuring that valuable historical documents are preserved for future generations. This study highlights the potential of VOC analysis through HS-SPME-GC/MS as a non-invasive tool for assessing the preservation state and degradation mechanisms of historical books. By examining VOC emissions from two books—a 16th-century Latin grammar and a 19th-century mathematics handbook—we identified distinct chemical profiles that reflect the impact of material composition and historical production methods on the longevity of paper-based artifacts.

The 16th-century book, constructed from high-quality rag-based paper, exhibited a VOC profile dominated by aldehydes and long-chain alkanes, indicative of oxidative degradation—a slower process that aligns with its durable material. In contrast, the 19th-century book, made from wood pulp, showed elevated furfural levels, a marker of acid-catalyzed hydrolysis, pointing to rapid degradation characteristic of acidic wood pulp paper. These differences underscore the need for tailored conservation strategies to mitigate the degradation of historically significant books, particularly those vulnerable to acid-catalyzed decay.

While the VOC profiles primarily reflect degradation of the paper substrate—namely, aldehydes from lipid oxidation and furfural from cellulose hydrolysis—minor contributions from inks, adhesives, or sizing agents cannot be fully excluded. No illustrations or visible ink corrosion were observed, and the detected VOCs did not include typical ink-related markers such as aromatic amines or phthalates [8,12,16,17]. Prior studies have reported compounds like isopropyl esters and dimethylformamide as possible indicators of inks or industrial additives in historical papers [16,17]. Thus, while ink degradation likely had minimal impact in the present analysis, future investigations using non-invasive techniques (e.g., FTIR, XRF, hyperspectral imaging) may help clarify the role of non-cellulosic components in VOC emissions [16,17].

In addition to degradation markers, compounds such as menthol, diphenyl ether, and diethyl phthalate were detected. These may reflect past preservation treatments or synthetic additives but could also result from environmental exposure or sample handling. Similar compounds have been reported in historical papers as possible contaminants or additives [8,12,13]. In the absence of direct historical or material evidence, we interpret these findings with caution.

Of note, this project was originally developed as part of a high school educational program, which shaped its exploratory and didactic focus. While scientific rigor was maintained in data acquisition and interpretation, some experimental details and long-term data storage practices typical of more advanced analytical workflows were beyond the intended scope. Nonetheless, we believe this study offers a valuable proof-of-concept and contributes meaningful insights into non-invasive diagnostic approaches for heritage materials, particularly in highlighting how accessible analytical techniques like HS-SPME-GC/MS can support paper-based conservation efforts.

Indeed, this project was made possible through a collaborative effort between public education and private industry, specifically involving teachers and students from IIS G. Torno (Castano Primo, Italy) and the heritage science researchers at InnovHub SSI (Milan, Italy). This partnership not only facilitated access to unique historical materials and advanced analytical techniques but also provided educational and hands-on scientific experiences for students. The collaboration exemplifies how interdisciplinary efforts between academic institutions and industry can enrich both scientific research and cultural preservation efforts.

By fostering such partnerships, we can continue to develop and refine non-invasive methods to support the long-term preservation of cultural heritage, ensuring that these artifacts remain accessible to future generations.

## 4. Materials and Methods

### 4.1. Sample Collection and Storage

Two historical books—a 16th-century Latin grammar by Gian Alberto Bossi and a 19th-century mathematics handbook, sourced from the Biblioteca Capitolare of Busto Arsizio in Italy—were selected for VOC analysis, according to [9,11]. Each book was stored in a 12 L glass desiccator, equipped with a silicone septum to accommodate the insertion of an SPME fiber. To minimize potential contamination, storage of the books was carried out in a new glass desiccator.

### 4.2. Headspace Solid-Phase Microextraction

The VOCs emitted were captured using HS-SPME. A 50/30 μm divinylbenzene/carboxen/polydimethylsiloxane (DVB/CAR/PDMS) fiber (Supelco, Bellefonte, PA, USA) was selected for its broad adsorption range, covering both polar and non-polar compounds. A new SPME fiber was used and preconditioned by heating it to 270 °C for 1 h to remove any potential contaminants, ensuring sample integrity. The same SPME fiber was then used to perform all the analyses (blanks and samples). The SPME fiber was preconditioned by heating it to 270 °C for 1 h to remove any potential contaminants, ensuring sample integrity. 

To enhance headspace VOC concentration, the books were positioned upright with their central pages opened and slightly fanned out to maximize surface exposure within the chamber. The selected pages exhibited the highest textual density; neither volume included illustrations or distinct ink formulations. The selected pages exhibited the highest textual density; neither volume included illustrations or distinct ink formulations. The HS-SPME extraction was carried out at 20 °C for 24 h. This duration was selected based on the procedure described in [10], where a 24-h extraction was demonstrated to yield reproducible and representative signals across a wide range of VOCs.

Prior to sampling, each desiccator was evacuated to remove any potential environmental contaminants and then sealed and left undisturbed for 24 h at an ambient temperature of 20 °C for a blank exposure.

### 4.3. Gas Chromatography–Mass Spectrometry Analysis

After VOC collection, the fiber was immediately inserted into an Agilent 7890B gas chromatograph coupled with a 5977B mass spectrometer (Agilent Technologies, Santa Clara, CA, USA) for compound desorption and analysis. The chromatographic separation was conducted using a polar polyethylene glycol (PEG) capillary column (50 m in length, 0.2 mm internal diameter, 0.4 μm film thickness), ideal for separating complex organic mixtures. The oven temperature was initially set at 40 °C and then gradually increased at a rate of 10 °C per minute until reaching 250 °C, where it was held for 5 min. Helium served as the carrier gas at a constant flow rate of 1.0 mL/min.

The mass spectrometer operated in electron ionization (EI) mode at 70 eV, scanning mass-to-charge ratios (*m*/*z*) from 40 to 450 amu to capture a comprehensive VOC profile. Identification was based on comparing observed spectra to the NIST Mass Spectral Library, version 2020 (National Institute of Standards and Technology, Gaithersburg, MD, USA), using a minimum spectral similarity index (SI) threshold of 600. Then, the compound identification is considered a correct identification if the maximum similarity score of a query mass spectrum fulfills this threshold. In order to improve the identification process, the linear retention indices (LRIs) were calculated. This latter parameter was calculated by injecting a mixture of linear alkanes, from C10 to C30, under the same instrumental conditions as the samples. A difference between the calculated and tabulated RLIs of about 50 units is considered acceptable, using a polar gas chromatographic column. The results obtained are shown in Table 1. Using SI and LRI simultaneously improves the accuracy of the library search, focusing particularly on aldehydes, furans, alkanes, and alcohols as primary markers of lipid oxidation and cellulose hydrolysis, indicative of the primary degradation pathways in historical paper.

### 4.4. Semiquantitative Analysis of VOCs

To compare VOC concentrations between the two books, semiquantitative analysis was performed. Each compound’s chromatographic peak area was integrated and expressed as a percentage of the total ion chromatogram. The results are shown in Table 1 as the average of two independent analyses on each book. The control of the duplicate analyses was evaluated on the basis of the relative areas %, which did not differ from each other by more than 10%. This relative quantification enabled the comparison of VOC abundances, offering insights into specific degradation processes. Furfural, a byproduct of cellulose hydrolysis, served as an indicator of acid-catalyzed degradation, predominantly observed in the 19th-century volume. In contrast, the 16th-century book showed higher levels of straight-chain aldehydes, such as octanal, nonanal, and decanal, which are typical products of lipid oxidation, suggesting a greater oxidative stability. These peak area percentages provided a comparative basis for assessing each book’s preservation state.

### 4.5. Quality Control and Blank Runs

Quality control was ensured by weekly monitoring of the empty desiccator using 24-h blank SPME fiber exposures to detect potential background contamination. Each fiber was reconditioned before sampling, and blank runs were conducted after each analysis to verify the absence of carry-over. These measures ensured the accuracy and reproducibility of the VOC profiles obtained.

## Figures and Tables

**Table 1:** Retention times (minutes), similarity index (SI) obtained using NIST-20 Mass Spectral Library, and theoretical linear retention index (LRI) (NIST-20 library) calculated by injecting a mixture of linear alkanes in the range C10-C30 under the same instrumental conditions and relative peak areas (%) of volatile organic compounds (VOCs) detected in the 16th-century Latin grammar book and the 19th-century mathematics handbook. The table highlights the presence of key compounds associated with oxidative degradation (e.g., aldehydes and alkanes) in the 16th-century book and acid-catalyzed hydrolysis markers (e.g., furfural) in the 19th-century book, reflecting distinct degradation pathways due to differences in paper composition and manufacturing techniques. Confidence in compound identification was ensured using a combination of spectral similarity index (SI) and comparison of theoretical and experimentally calculated linear retention indices (LRIs). VOC analysis was conducted in duplicate for each book, and Table 1 reports the average relative peak areas (%). To evaluate meaningful differences between the degradation processes of the two books, total aldehyde, total hydrocarbon, and furfural values were compared using a two-tailed *t*-test. All three parameters showed statistically significant differences at the 95% confidence level (*p* < 0.05) and are marked with an asterisk (*).

	Retention Time (min)	SI (NIST-20)	LRI (Calculated)	LRI (Theoretical)	Area %	
***Compound***					***XVI Century Book***	***XIX Century Book***
Octanal	17.15	771	1304	1289 (±9)	0.42	0.46
Tetradecane	18.98	909	1401	1400	12.48	17.81
Nonanal	19.36	879	1424	1391 (±8)	2.36	2.25
Pentadecane	20.91	786	1502	1500	0.97	1.05
2-ethylhexanol	21.01	844	1506	1491 (±5)	1.61	1.57
Furfural	21.09	830	1511	1461 (±11)	0.11	0.31 *
Decanal	21.42	879	1538	1498 (±8)	4.76	3.90
Hexadecane	22.79	913	1601	1600	10.45	10.06
Menthol	24.05	911	1673	1637 (±6)	4.67	6.30
Octadecane	26.19	825	1802	1800	1.66	1.38
Butoxyethoxy ethanol	26.73	894	1827	1796 (±13)	4.35	0.71
Anethole	27.49	698	1845	1817 (±5)	1.05	1.43
Diphenyl ether	30.61	809	2129	2017 (±15)	1.01	1.42
Diisopropylnaphthalene	32.32	719	2230	2228 (±14)	4.65	5.06
Diethyl phthalate	35.58	745	2394	2365 (±7)	0.77	1.05
Benzophenone	38.27	678	2508	2450 (±16)	0.59	0.36
Diisobutyl phthalate	38.49	916	2525	2536 (±10)	5.76	1.45
Total aldehyde					7.54 *	6.61
Total hydrocarbons					25.56	30.30 *

## Data Availability

The data supporting the findings of this study are available from the corresponding author upon reasonable request. Due to the educational scope of the project and the lack of ongoing access to the original historical books, supplementary files such as full chromatograms, match score tables, or raw GC-MS signal data were not archived for external sharing.

## Appendix A

The 5B IISS Torno Working Group.

Susanna Andrani, Simone Barzizza, Samuele Busti, Pietro Carturan, Luca Casucci, Mathias Chiodini, Noemi Freddi, Micaela Gallazzi, Andrea Maino, Sara Menescardi, Federica Milanti, Alessandro Pagano, Alessio Perlini, Mattia Rizzo, Davide Valente.
